# Using Deep Learning to Forecast Maritime Vessel Flows

**DOI:** 10.3390/s20061761

**Published:** 2020-03-22

**Authors:** Xiangyu Zhou, Zhengjiang Liu, Fengwu Wang, Yajuan Xie, Xuexi Zhang

**Affiliations:** 1 Navigation College, Dalian Maritime University, Dalian 116026, China; 2 Centre for Maritime Studies, National University of Singapore, Singapore 118414, Singapore; 3 Department of Industrial Systems Engineering and Management, National University of Singapore, Singapore 117576, Singapore; 4 School of Automation, Guangdong University of Technology, Guangzhou 510006, China; zxxnet@gdut.edu.cn

**Keywords:** maritime vessel flows, intelligent transportation systems, deep learning

## Abstract

Forecasting vessel flows is important to the development of intelligent transportation systems in the maritime field, as real-time and accurate traffic information has favorable potential in helping a maritime authority to alleviate congestion, mitigate emission of GHG (greenhouse gases) and enhance public safety, as well as assisting individual vessel users to plan better routes and reduce additional costs due to delays. In this paper, we propose three deep learning-based solutions to forecast the inflow and outflow of vessels within a given region, including a convolutional neural network (CNN), a long short-term memory (LSTM) network, and the integration of a bidirectional LSTM network with a CNN (BDLSTM-CNN). To apply those solutions, we first divide the given maritime region into M×N grids, then we forecast the inflow and outflow for all the grids. Experimental results based on the real AIS (Automatic Identification System) data of marine vessels in Singapore demonstrate that the three deep learning-based solutions significantly outperform the conventional method in terms of mean absolute error and root mean square error, with the performance of the BDLSTM-CNN-based hybrid solution being the best.

## 1. Introduction

Forecasting traffic flows of vessels has been recognized as a challenging task in the maritime intelligent transportation system, since it could be affected by various complex factors [1,2]. Accurate and timely traffic information is of significant importance to both maritime managers and individual vessels, as it not only helps the former to better conduct port planning [3,4], alleviate congestion [5,6], mitigate emission of GHG (greenhouse gases) and improve public security [7,8], but also enables the latter to better operate the ship navigation system and plan a route [9,10], so as to avoid collisions and reduce the potential cost due to late arrival [11].

Various solutions have been studied for forecasting maritime traffic flow. Wang et al. [12] developed a vessel flow prediction model using a back propagation (BP) neural network, in which training and data sampling were conducted based on a residual analysis. Haiyan and Youzhen [13] came up with a hybrid scheme for vessel flow forecasting, which integrated an RBF (radial basis function) neural network, grey forecasting and auto-regression into a framework of support vector regression (SVR). The experimental results based on a real dataset demonstrated that the combined method significantly outperformed each individual. Li et al. [14] conceived a hybrid method centering around a robust support vector regression (RSVR) model. In that method, a chaotic cloud simulated annealing genetic algorithm was first developed to optimize the parameters of RSVR, then the kernel principal component analysis (KPCA) algorithm was utilized to finalize the input vectors. Afterwards, the hybrid framework was established, and the test based on a real dataset justified its advantages over others. Yang et al. [15] developed an enhanced hybrid RBF neural network (EHRBF-NN) to forecast the traffic flow of vessels. The method was robust in that it incorporated a regression tree and a particle swarm optimization (PSO) algorithm into the RBF neural network. More specifically, the regression tree was exploited to calculate the parameters (i.e., centers and radius) of RBF, and the PSO was designated to address the issue of potential over-fitting and update the weights of the neural network. Liu et al. [16] proposed a two-step method to predict vessel flows. Particularly, in the first step, they separated vessel traffic flow into low-rank and sparse components by adopting a non-convex model. Then in the second step, the ARIMA (auto-regressive integrated moving average) and WNN (wavelet neural network) were explored to predict the flows based on the low-rank and sparse components from the fist step. He et al. [17] proposed an improved Kalman model for short-term vessel traffic flow prediction. In that model, a preliminary prediction was first done using a regression analysis, which was then adopted to replace the transfer equation of the Kalman filter. Xiao et al. [11] came up with a probabilistic method for maritime traffic forecasting. The method was novel in that it took both waterway pattern knowledge and vessel motion stability into account. In particular, the waterway pattern was learnt via a lattice-based DBSCAN algorithm which also helped narrow down the problem scale, and the motion behavior was modeled through a kernel density estimation, thus, the combination of them was supposed to successfully predict the traffic flow.

Although some successes have been achieved in maritime traffic flow forecasting, most of the methods above are shallow and outdated to some extent, given the fact that the deep learning approach has been unarguably deemed as the state-of-the-art in many areas, such as computer vision, natural language processing, and transportation. The promising advantages brought by deep learning can be summarized as two aspects: (1) deep neural networks can be designed to solve end-to-end tasks, which directly takes the raw data as input without elaborating the intermediate feature extraction; (2) it usually achieves significantly higher accuracy in comparison with conventional methods. Despite the fact that numerous deep learning approaches have been applied in traffic flow or traffic condition forecasting, most of them focus on the land transportation instead of maritime. Lv et al. [18] proposed a deep learning framework to predict the freeway traffic flow, by exploring the big data from road infrastructure sensors. At the heart of that approach was a stacked auto-encoder (SAE) model, which was adopted to learn the latent traffic flow features by inherently taking spatial and temporal correlations into account. Chen et al. [19] implemented a typical stacked LSTM (long short-term memory) network to forecast the traffic congestion, which was applied to a realistic navigation map. The results demonstrated that the proposed approach was able to effectively learn the hidden patterns of traffic flows. Cui et al. [20] proposed a deep stacked bidirectional and unidirectional LSTM (SBU-LSTM) network to predict the traffic speed, which considered both forward and backward dependencies of the traffic data. More specifically, in that approach, a bidirectional LSTM (BDLSM) layer was developed to learn the spatial features and bidirectional temporal relationships by exploiting the historical data, as such it was able to grasp the dynamic nature of the traffic. Polson and Sokolov [21] came up with a deep learning architecture to predict the short-term traffic flow. Particularly, that architecture integrated a linear model with $\ell_{1}$ regularization and a branch of tanh layers, the first layer of which was designed to identify the spatio-temporal relations among predictors and other layers. As a consequence, it was able to capture the sharp nonlinear patterns of the traffic flow. Yu et al. [22] designed a spatio-temporal deep neural network to predict the short-term traffic speeds. Enlightened by the research outcomes of motion detection in the field of computer vision, they first segmented the road network into grids, then the traffic information inside each grid was input to a convolutional neural network (CNN) to learn the spatial relationship. After that, the output of which was further taken as the input of a LSTM network, to capture the temporal relationship. Zhang et al. [23] proposed a deep spatio-temporal residual network (ST-ResNet) to forecast the citywide crowd inflow and outflow. In that approach, a residual neural network framework was first adopted to model the temporal closeness, period, and trend properties of the crowd flows, then a sequence of residual convolutional units were designed to model the spatial relationships of crowd flows, and lastly, the output of those residual neural networks were aggregated as the preceding layer to the final output layer of real crowd flows. Guo et al. [24] presented a framework for predictor fusion to forecast the short-term traffic condition. In the framework, three strategies were employed to evaluate the fusion performance, i.e., average fusion, weighted fusion and kNN fusion. Experimental results based on real dataset verified the significant advantages of the fusion method over the stand-alone ones. Liu and Chen [25] developed a novel prediction model for passenger flow using deep learning approach. The core of the model was the combination of a pre-trained unsupervised SAE with a supervised deep neural network, which was supposed to well extract the hierarchical features, so that the passenger flow for any periods from Monday to Sunday could be successfully forecast. Ke et al. [26] devised a deep learning approach to predict the road traffic congestion. The approach was distinguished in that it utilized visual signals to learn the traffic flow, and moving object detection and moving speed calculation were done by integrating a CNN and a Gaussian mixture model (GMM).

Obviously, the applications of deep learning in land transportation have been investigated far more extensively than that of maritime. On one hand, large-scale data for the land transportation is not that hard to obtain compared with maritime transportation, which is the foundation of applying the deep learning techniques. On the other hand, unlike the vessels in the sea, the mobilities of cars or people on land are mostly restricted to streets or rails, therefore, the traffic pattern for which is comparatively easy to be revealed. Moreover, we would like to note that, a variety of research with respects to the deep learning in maritime have been conducted, however most of them focus on vessel type identification [27], trajectory prediction or reconstruction [28], anomaly detection [29,30] and collision avoidance [31,32,33]. The problem of how to adopt deep learning to forecast the traffic flows for maritime has not yet been studied.

Therefore, in this paper, centering around the deep learning approach, we propose three different solutions to forecast the inflow and outflow of vessels within a given marine region. More specifically, the three solutions are featured by a CNN, a LSTM network, and the integration of a bidirectional LSTM network with a CNN (BDLSTM-CNN), respectively. In particular, with respects to the BDLSTM-CNN based solution, each input will be first fed into the convolutional layers, then they will go through the forward layer and backward layer of a bidirectional LSTM network. As such, this hybrid solution is supposed to coherently learn the spatial and temporal dependencies pertaining to the vessel flows. Moreover, to apply the three deep learning based solutions into practice, we divide the given marine areas into M×N grids. Then all the solutions are implemented to forecast the inflow and outflow for each grid. Experimental results based on the real AIS data for a given area in Singapore show that the three deep learning based solutions significantly outperform the conventional method, in which the hybrid solution BDLSTM-CNN achieves the best performance.

The remainder of the paper is organized as follows. Section 2 introduces the preliminaries first and then elaborates the structures and logic of the three deep learning based solutions. Section 3 presents the comprehensive experimental results and analysis. Section 4 concludes the paper and states the future works.

## 2. Deep Learning Based Solutions

In this section, we first introduce the preliminaries regarding the vessel flow forecasting problem. Then we present three deep learning based solutions, i.e., CNN, RNN, and BDLSTM-CNN, respectively.

### 2.1. Preliminaries

**Figure 1:**
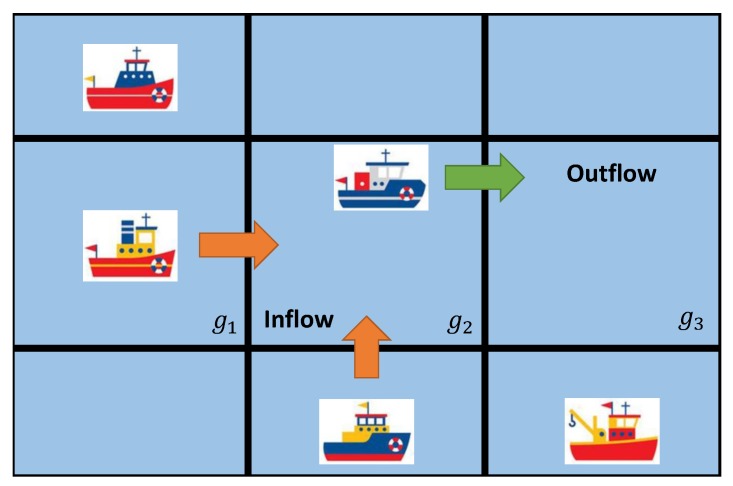
Illustration of the Inflow and Outflow.

Our target is to forecast the inflow and outflow of vessels within a given area. To this end, we first divide this area into M×N grids. Thus, the inflow and outflow at a time step can be represented by two matrix, respectively, and the element of which refers to the counts of vessels entering or leaving the corresponding grid during that time step. In this regard, our task comes down to predict the inflow matrix and outflow matrix using the historical traffic flow data. Formally, given the historical observations $\left\{X_{t}|t=0,1,\ldots ,n-1\right\}$, our task is to predict $X_{t+\tau }$, where τ≥1 and is an integer. In particular, $X_{t}=\left[I_{t},O_{t}\right]$, where $I_{t}$ and $O_{t}$ represent the inflow matrix and outflow matrix at time step *t*, respectively. Taking Figure 1 as an example, at time step *t*, two vessels will enter grid $g_{2}$, and one vessel will leave, then $I_{t}\left(g_{2}\right)$ = 2, and $O_{t}\left(g_{2}\right)$ = 1, respectively.

### 2.2. CNN Based Solution

**Figure 2:**
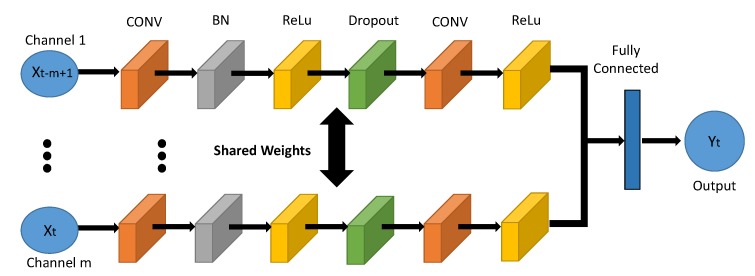
Illustration of the CNN based Solution.

Convolutional neural network has been successfully applied in many areas, such as video analysis [34] and transportation [35], which is characterized by its strong capability of capturing the spacial representation. CNN can be employed here to handle the traffic flow prediction problem for two reasons. On one hand, the flow of vessels in a grid could be affected by the neighbor or distant grids, and CNN has the potential to capture this dependency, as the convolution operation inside CNN somehow can help predict the movement of vessels from the spatial perspective. On the other hand, the inflow and outflow for the whole given area are represented as matrix in our problem, which is the most inherent format of inputs to CNN. And the structure of the proposed CNN-based solution is depicted in Figure 2.

Accordingly, this CNN based solution has *m* channels, which correspond to the *m* historical observations as the inputs. Meanwhile, all the channels share the same weights. In particular, each input contains both the inflow and outflow matrix, and each input channel includes two convolutional layers, two ReLu layers, one batch normalization (BN) layer, and one dropout layer. Among them, the convolutional layers play the most important role, which are used to learn spacial features of different levels [22]. The underlying logic of convolutional layer is expressed as follows:

(1) $$o_{r}^{l}=\sum_{k}W_{kr}^{l}*o_{k}^{l-1}+b_{k}^{l},$$

where $o_{r}^{l}$ refers to the output of the $r_{th}$ filter in the $l_{th}$ layer; $o_{k}^{l-1}$ refers to the output of the $k_{th}$ filter of the preceding layer; $W_{kr}^{l}$ and $b_{k}^{l}$ refers to the weights and bias; ∗ refers to the convolution operation. Besides, the BN layer is used to scale the range of feature values; the ReLu layer acts as the activation function; the Dropout layer is used to select the salient features from the receptive region, so as to avoid redundant features and reduce the scale of the computation; and the output layer generates both inflow and outflow matrix that we would like to predict. To adopt the CNN based solution to solve the vessel flow forecasting problem, we take $X_{t-m+1}$ to $X_{t}$ as the inputs at time step *t* and $X_{t+\tau }$ as the output $Y_{t}$.

### 2.3. LSTM Based Solution

Due to the capability of sequential and temporal modeling, recurrent neural network (RNN) also has been successfully applied to many challenging practices, such as natural language processing, stock forecasting and crowd density prediction [36]. However, traditional RNN sometimes suffers from the issue of vanishing and exploding gradient if the learning sequence is long. To address this issue, a variant of RNN, termed LSTM (Long short-term memory) model, was devised, which utilized memory cells with various gates to preserve useful information for long-term dependencies [37]. In view of the fact that the vessel flow for a given area can be considered as the classical time series with temporal dependency, the LSTM network is supposed to well capture the temporal correlations in the flow forecasting problem.

**Figure 3:**
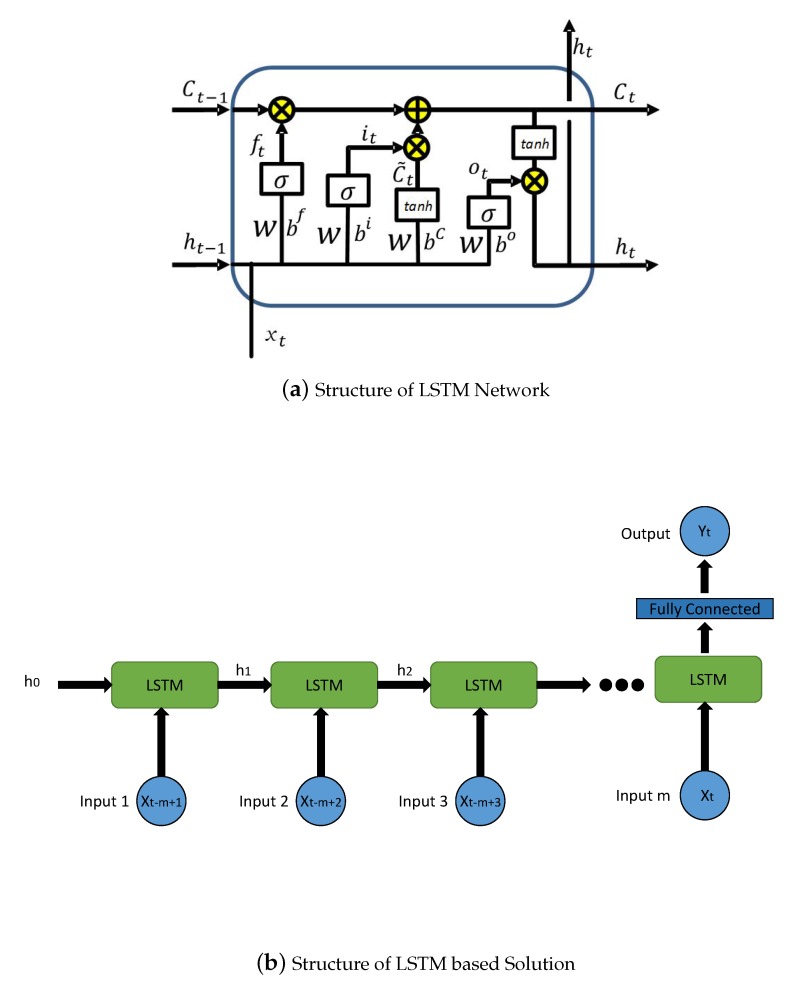
Structure of the LSTM network, and the LSTM based Solution.

More specifically, the structure of a single layer LSTM network is depicted in Figure 3a. And the LSTM network updates itself at time step *t* as follows [38]:

(2) $$\begin{array}{cc} \; & f_{t}=\sigma \left(W^{f}\left[h_{t-1},x_{t}\right]+b_{f}\right)\\ \; & i_{t}=\sigma \left(W^{i}\left[h_{t-1},x_{t}\right]+b_{i}\right)\\ \; & {\tilde{C}}_{t}=tanh\left(W^{C}\left[h_{t-1},x_{t}\right]+b_{C}\right)\\ \; & C_{t}=f_{t}*C_{t-1}+i_{t}*{\tilde{C}}_{t}\\ \; & o_{t}=\sigma \left(W^{o}\left[h_{t-1},x_{t}\right]+b_{o}\right)\\ \; & h_{t}=o_{t}*C_{t}, \end{array}$$

where $h_{t}$ is the hidden state; $i_{t},f_{t}$ and $o_{t}$ refer to the input gate, forget gate and output gate, respectively; ${\tilde{C}}_{t}$ and $C_{t}$ refer to the input modulation gate and memory gate, respectively. $\left\{W^{f},W^{i},W^{C},W^{o}\right\}$ are the weights, and $\left\{b^{f},b^{i},b^{c},b^{o}\right\}$ are the biases for the corresponding gates; σ(·) and tanh(·) are sigmoid and hyperbolic tangent activation functions, respectively. The memory cell unit $C_{t}$ contains two components, i.e., previous memory cell unit $C_{t-1}$ modulated by $f_{t}$ and $\tilde{C}$, which is modeled by the current input, and previous hidden state, modulated by the input gate $i_{t}$ [38]. The essence of sigmoidal operation for $i_{t}$ and $f_{t}$ normalizes themselves into the scope of [0,1]. Particularly, they could be deemed as knobs that LSTM learns to selectively forget its previous memory or consider its current input. In a similar way, the output gate $o_{t}$ models the transfer from memory cells to hidden states. On the basis of these mechanisms, the LSTM network is supposed to learn complex and temporal dynamics that exist in sequential vessel movement measurements, engendering a satisfactory performance for vessel flow forecasting.

With the fundamental logic of the LSTM network, we build up the complete structure of the LSTM based solution, which is depicted in Figure 3b. In this solution, there are *m* inputs at the time step *t*, i.e., $X_{t}$, $X_{t-1}$, …, $X_{t-m+1}$, all of which are fed into the LSTM networks in order. Afterwards, the outputs of the LSTM network are connected to a fully connected network layer, then $Y_{t}$ is regarded as the final output, which is set as $X_{t+\tau }$.

### 2.4. BDLSTM-CNN Based Hybrid Solution

As we have stated previously, the vessel flow forecasting relies on both spatial and temporal dependencies. Therefore, it would be desirable to integrate the CNN and the LSTM network into a comprehensive framework, to coherently learn the spatiotemporal relationships for the vessel flow forecasting. However, with respect to the temporal feature learning, we exploit a more powerful recurrent neural network, namely, bidirectional LSTM, to replace the traditional unidirectional LSTM.

#### 2.4.1. Bidirectional LSTM

**Figure 4:**
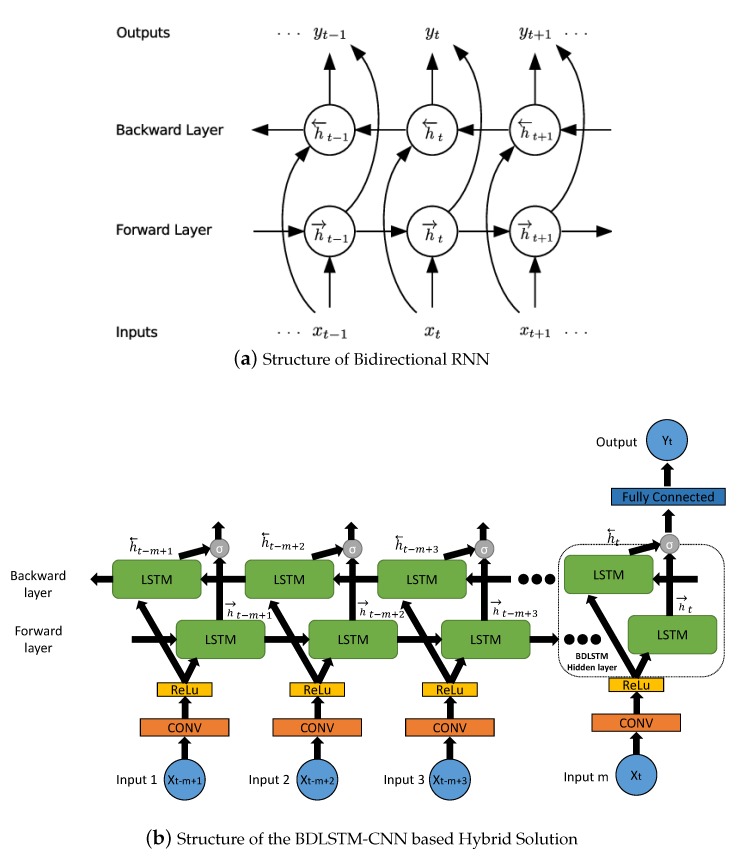
Structure of Bidirectional RNN, and the Hybrid Solution.

The idea of BDLSTMs is derived from the bidirectional RNN, which is used to address a crucial issue that, the conventional RNN is only able to make use of the previous context, thus they only learn representations from previous time steps [39]. However, we might have to learn representations from future time steps to better understand the context and eliminate ambiguity sometimes. As a consequence, the bidirectional RNN was developed to achieve this goal, which processed sequence data in both forward and backward directions with two separate hidden layers. And both of them are connected to the same output layer, as depicted in Figure 4a. More specifically, the bidirectional RNN separates the hidden layer into two parts, forward state sequence $\vec{h}$ and backward state sequence $\overset{\leftarrow }{h}$, and they are computed as follows [40]:

(3) $$\begin{array}{cc} \; & {\vec{h}}_{t}=H\left(W_{x\vec{h}}x_{t}+W_{\vec{h}\vec{h}}{\vec{h}}_{t-1}+b_{\vec{h}}\right)\\ \; & {\overset{\leftarrow }{h}}_{t}=H\left(W_{x\overset{\leftarrow }{h}}x_{t}+W_{\overset{\leftarrow }{h}\overset{\leftarrow }{h}}{\overset{\leftarrow }{h}}_{t+1}+b_{\overset{\leftarrow }{h}}\right)\\ \; & y_{t}=W_{\vec{h}y}{\vec{h}}_{t}+W_{\overset{\leftarrow }{h}y}{\overset{\leftarrow }{h}}_{t}+b_{y}. \end{array}$$

Accordingly, the deep bidirectional RNN can be established by stacking multiple RNN hidden layers on top of each other. Each hidden state sequence, $h^{n}$, is replaced by the forward and backward, $\vec{h}{}^{n}$ and $\overset{\leftarrow }{h}{}^{n}$. On the other hand, deep bidirectional LSTM (BDLSTM) can be achieved based on the integration of deep bidirectional RNN and the LSTM network. Thus, the BDLSTM is supposed to have the ability to model the deep representation of long-span features [39].

#### 2.4.2. The Hybrid Solution

With the framework of bidirectional LSTM, CNN is further integrated to build up a hybrid solution, which is depicted in Figure 4b. In this framework, each input first goes through a convolution layer and a Relu layer, to learn the spatial dependency. Then the output will be fed into the bidirectional LSTM network (i.e, the dotted box in Figure 4b), as the input for both forward sequence and backward sequence. Afterwards, the outputs of the bidirectional LSTM hidden layers are further connected to a sigmoid activation function, which is followed by a fully connected layer. Lastly, the final output is regarded as the predicted vessel flow.

To summarize from a high level perspective, at each time step *t*, there are *m* inputs that will be fed into the synthesized networks, i.e., $X_{t-m+1}$, $X_{t-m+2}$, …, and $X_{t}$, each of which consists of both inflow matrix and outflow matrix. At the same time, the output $Y_{t}$ is set as $X_{t+\tau }$. Normally, τ is equal to 1, however it can also take much larger integer values. On the other hand, since the vessel flow forecasting is a regression task in nature, root mean square error or mean absolute error are usually adopted as the loss function. However, in this hybrid solution, we consider a new loss function for final objective optimization, namely, smooth $\ell_{1}$ loss [41], which is expressed as follows:

(4) $$loss\left(x,y\right)=\left\{\;\begin{array}{c} 0.5{\left(x-y\right)}^{2},\;\;if\;\left|x-y\right|<1;\\ |x|-0.5,\;\;otherwise. \end{array}\right.$$

Smooth $\ell_{1}$ loss, also called Huber loss, is usually less sensitive to abnormal inputs, and also helps the networks prevent gradient exploding to some extent [41].

## 3. Experimentation and Evaluation

In this section, we conduct experimentation in different settings to test the proposed solutions, and demonstrate their advantages over the baseline. Particularly, we first introduce the data processing and experimental settings, then we compare and evaluate the three deep learning based solutions. Finally, we compare the three solutions with a conventional method, i.e., support vector regression (SVR).

### 3.1. Data Processing and Experimentation Setup

**Figure 5:**
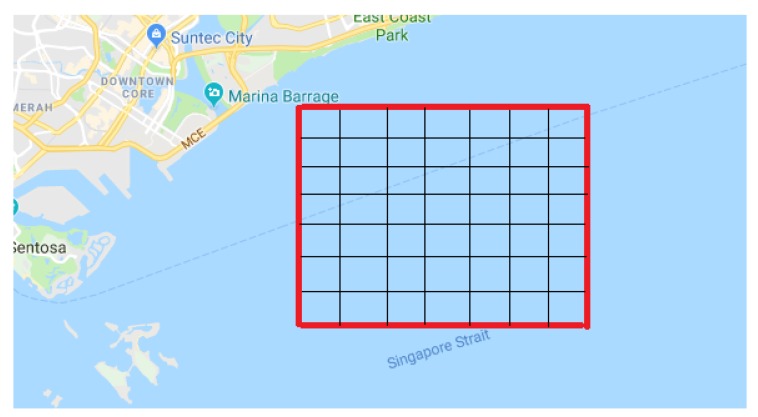
The Selected Marine Area: 7×7 Grids.

We use the AIS (Automatic Identification System) data of maritime vessels to perform the forecasting task, which is an automatic tracking system that uses transponders on ships to generate trajectory-related information [30]. The AIS data for a vessel contains many useful attributes regarding its movement and mobility, which can be obtained from https://www.marinetraffic.com. Here we mainly exploit the information of vessel ID, coordinates (i.e., longitude and latitude) and time stamp. The testing filed we selected is an rectangle marine area southeast to Singapore, which is shown as the red rectangle in Figure 5. The location of the left upper point is ($1.287979^{{}^{\circ}}$, $103.892723^{{}^{\circ}}$), and that of the right lower point is ($1.235027^{{}^{\circ}}$, $103.996817^{{}^{\circ}}$). In all the experimentation we conducted, we uniformly divided this area into 7×7 grids (i.e., M=N=7), which means that both inflow matrix $I_{t}$ and outflow matrix $O_{t}$ have a size of 7×7.

Then we process and analyze the AIS data, and identify all the vessels entering and leaving those grids accordingly. Particularly, the AIS data we collected for the given area lasts about 31 days, from 01-10-2013 to 31-10-2013. We set the duration for each time step as 5 min. Then we have 8525 samples for each grid (with some data missed), which include the amount of vessels entering and leaving the given grid. We divide them into training dataset and testing dataset according to the time order, i.e., from 01-10-2013 to 25-10-2013, and from 26-10-2013 to 31-10-2013, which include 6305 and 2220 samples, respectively. In addition, we implement all the experimentation using pytorch, in a laptop with Intel i7 CPU, 8G RAM, and Nvidia GTX 1060.

### 3.2. Error Performance for the Deep Learning Based Solutions

**Figure 6:**
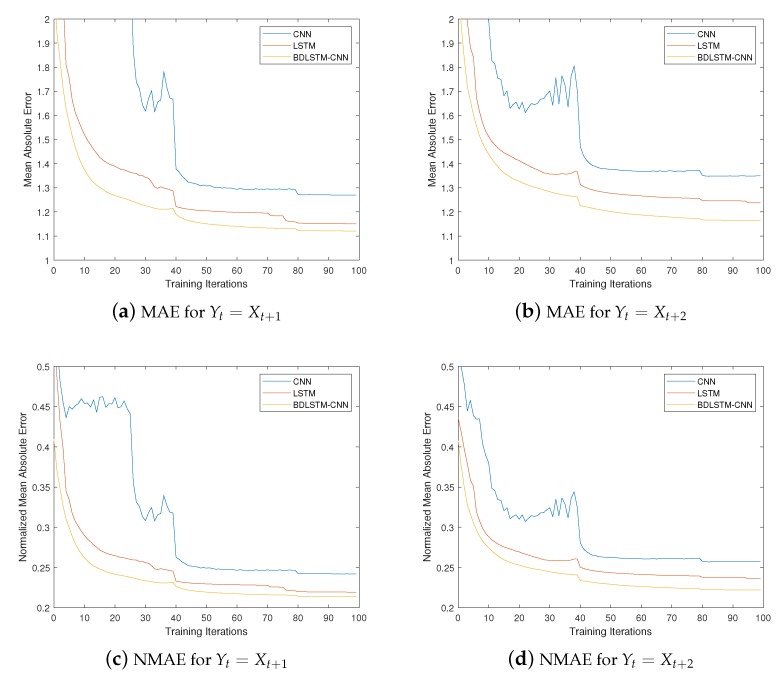
Performance Regarding MAE and NMAE.

**Figure 7:**
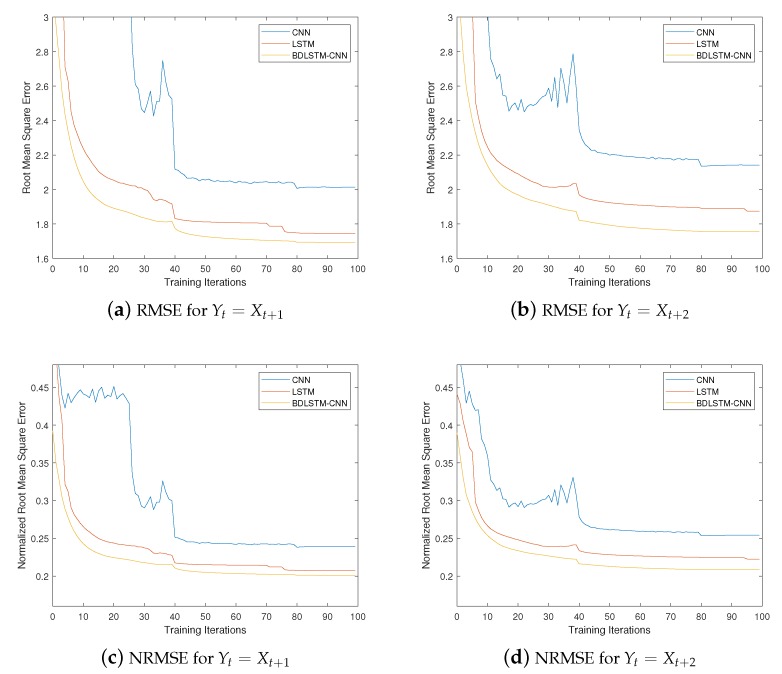
Performance Regarding RMSE and NRMSE.

We set $Y_{t}=X_{t+1}$ and $Y_{t}=X_{t+2}$, respectively, and conduct experimentation according to the above configurations. We use two types of errors to measure the performance of the proposed solutions, i.e., mean absolute error (MAE) and root mean square error (RMSE), since they are the two most important metrics for regression problems. We also use their normalized forms to further evaluate the performance, i.e., NMAE and NRMSE. All the results for the three deep learning based solutions are shown in Figure 6 and Figure 7. Before looking into them, we would like to note that the errors in those figures are calculated by considering inflow and outflow together. Additionally, all the results below are obtained based on the testing data.

From Figure 6a,b we can see that, as the training iteration increases, the MAE for testing data of the three deep learning based solutions drop quickly. With respect to both $Y_{t}=X_{t+1}$ and $Y_{t}=X_{t+2}$, all the solutions seem to converge after about 85 iterations. Pertaining to the two different $Y_{t}$ settings, the BDLSTM-CNN based solution always achieves the lowest MAE, which are around 1.11 and 1.14, respectively. In contrast, the LSTM based solution achieves the second lowest MAE of 1.15 and 1.25, and the CNN based solution achieves the third lowest MAE of 1.29 and 1.35. It makes sense that the BDLSTM-CNN based hybrid solution engenders the best results as it coherently explores the spatial and temporal dependencies regarding the vessel flows, by integrating both CNN and LSTM. Moreover, with respect to the temporal expression, it exploits the bidirectional LSTM to take advantage of both past and future information, which is supposed to get better results than the unidirectional LSTM. Comparing the CNN based solution and the LSTM based solution, we can observe that, the latter presents a better performance, which is probably due to that the temporal relationship is much relatively important in comparison with the spatial relationship in this case. Although that the former solution takes every five preceding flow matrix to generate the succeeding one, and the convolution layer also has the potential to predict the movement of vessels from the spatial perspective, the structure of CNN itself does not have a scheme to capture the temporal correlation. This also might be able to explain the oscillation in the curve of CNN based solution, because it dose not have any mechanism to capture the seasonality pattern in the vessel flows. On the other hand, there are only slight oscillations for LSTM based solution and BDLSTM-CNN based solution, which justifies their favorable capability of handling the seasonality in sequence. Comparing Figure 6a,b, we can observe that, the performance of the three solutions slightly deteriorates for $Y_{t}=X_{t+2}$. It is reasonable as one can always make a more accurate forecast for a near future than for a far future. However, the hybrid solution still achieves the lowest error. Additionally, we would like to highlight that, the difference between the LSTM based solution and the hybrid solution is larger for $Y_{t}=X_{t+2}$ than that of $Y_{t}=X_{t+1}$. It happened because in the case of $Y_{t}=X_{t+2}$, the bidirectional LSTM may bring more useful information from the backward layer, in comparison with the solo forward layer in the LSTM based solution.

From Figure 6c,d, we can see that they share similar pattern with Figure 6a,b. This is normal because they are simply the normalized version of the latter, which can be regarded as a kind of error rate. The difference in the curve shape comes from the fact that we only show part of errors in Figure 6a,b, in order to highlight the region of interests. Looking into Figure 6c,d, we can observe that, the three solutions achieve error rates (in terms of MAE) of around 24.5%, 22.5% and 22.0%, respectively, for $Y_{t}=X_{t+1}$, and around 26%, 24% and 22.5%, respectively, for $Y_{t}=X_{t+2}$, with the hybrid solution being the lowest. The error rates in terms of MAE would be decreased about 1.5%, 1.5% and 0.5%. So it can be concluded that the results of three deep learning based solutions considerably are better than the traditional approach expressed in MAE, with the performance of the BDLSTM-CNN based hybrid solution being the smallest.

Likewise, we also use the RMSE (root mean square error) to further evaluate the proposed solutions, and the results are depicted in Figure 7, which share similar pattern with that of Figure 6. From Figure 7a,b, we can observe that, the three solutions achieve a RMSE of around 2.01, 1.75 and 1.68 for $Y_{t}=X_{t+1}$, respectively, and around 2.18, 1.88 and 1.75 for $Y_{t}=X_{t+2}$, respectively, with the hybrid solution being the lowest for both cases. From Figure 7c,d, we can see that, the three solutions achieve error rates (in terms of RMSE) of around 24%, 21% and 20%, respectively, for $Y_{t}=X_{t+1}$, and around 26%, 23.5% and 21%, respectively, for $Y_{t}=X_{t+2}$, with the hybrid solution being the lowest for both cases. Similarly, the error rates in terms of RMSE will be lowered by 2%, 2.5% and 1%. Consequently, it can be found that the outcomes of three deep learning based solutions noticeably outperformed than the traditional approach in terms of RMSE, with the performance of the BDLSTM-CNN based hybrid solution being the lowest.

Combining the performance of both MAE and RMSE in Figure 6 and Figure 7, it can be possibly proving that the BDLSTM-CNN based hybrid solution outperforms the CNN based solution and the LSTM based solution. However, considering that, (1) all the solutions only adopt five historical data to predict a new one, (2) all the errors are derived based on integrating inflow and outflow together, we believe that the performance of all the solutions are sufficiently satisfactory, although superiority and inferiority exist among them.

### 3.3. Breakdown Performance for the Hybrid Solution

Since the BDLSTM-CNN based solution has the best overall performance, we look into this hybrid solution and analyze its prediction capability in a breakdown perspective. To this end, we plot the curves of the average forecasting value of the inflow and outflow for the whole given region, and the forecasting value of inflow and outflow for a given grid, respectively. We would like to note that, all the results below are obtained based on the testing data.

**Figure 8:**
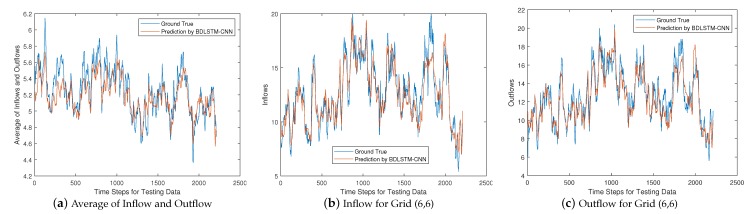
Prediction for $Y_{t}=X_{t+1}$.

We first plot the above forecasting values against the ground truth for $Y_{t}=X_{t+1}$ in Figure 8. From Figure 8a we can see that, the ground truth value of the average by considering inflow and outflow together, changes dramatically as time goes on. However, the BDLSTM-CNN based solution can well capture those trends, such as the sharp changes at time step 100, 1300, 1700 and 2200, respectively, because this hybrid solution combines the advantages of the capability of CNN and BDLSTM, to learn the spatial and temporal features in a unified way. Nevertheless, we observe some imperfect tracking, such as time step 250, which does not catch the crest. However, considering that the ground truth is the average of both inflow and outflow for the whole given area, some minor errors are tolerable. We also plot the forecasting curves of a single grid for inflow and outflow in Figure 8b,c, respectively, by taking grid (6,6) as an example. From Figure 8b, we observe that, the hybrid solution can catch most of the crests and troughs for the inflow curve, no matter how sharp or smooth they are, such as the crest at time step 400 and the trough at time step 1250. An unsatisfactory forecasting is found as well at time step 1800, where a sharp crest is missed. From Figure 8c, we observe that the hybrid solution can also successfully follow both sharp and smooth crests and troughs for the outflow curve, such as the crest at time step 1050, and trough at time step 1450. Although the hybrid solution mismatched the ground truth at some points, such as time step 1800, most of the failures are tolerable.

**Figure 9:**
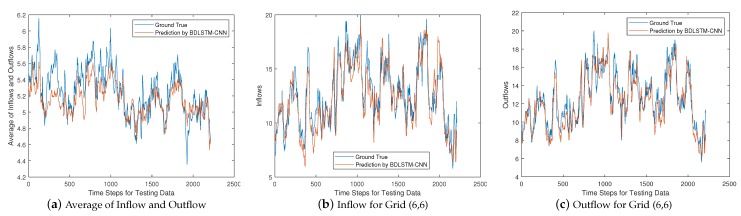
Prediction for $Y_{t}=X_{t+2}$.

We then continue to plot the forecasting values against the ground truth for $Y_{t}=X_{t+2}$ in Figure 9. From Figure 9a we can see that, the hybrid solution is still able to basically track the ground truth value, although slight deterioration is observed in comparison with the performance in Figure 8a. This can be explained by the fact that, a near future is comparatively easier to be forecast than a distant future. Despite the slight deterioration in the average forecasting, the hybrid solution still shows competitive performance for inflow and outflow forecasting pertaining to grid (6,6), which mostly well captures all the sharp or smooth crests and troughs.

Combining the results in both Figure 8 and Figure 9, we can conclude that the hybrid solution has strong capability of forecasting the inflow and the outflow of vessels. Though the inflow and outflow changed dramatically, the trend can be well captured by the hybrid solution.

### 3.4. Comparison with the Conventional Method

In this subsection, we compare the performance of the three deep learning based solutions with a conventional method, namely the support vector regression (SVR) based approach. In particular, we use the python sklearn package to implement this function, and utilize the same training dataset to optimize its parameters, and then do the evaluation using the same testing dataset, the results of which are recorded in Table 1. Since the normalized errors reflect a kind of error rate in forecasting, we mainly concentrate on the normalized forms of the two errors, i.e., NMAE and NRMSE.

From Table 1 we can see that, the error rates of the SVR method are around 51% for both measurements of MAE and RMSE, almost twice as much as the three deep learning based solutions. The inferiority comes from the fact that, the SVR method does not have a sophisticated scheme to learn the underlying spatial dependency, or the long-term temporal dependency. In contrast, CNN has a convolution layer, and LSTM or BDLSTM has a memory and gate to handle the two situations [42,43], respectively. On the other hand, the BDLSTM-CNN based hybrid solution always achieves the lowest error rates of 20% to 22.5%, which implies a forecasting accuracy of 77.5% to 80%. Considering that we only use five historical data to predict a new one, and both inflow and outflow of vessels change dramatically, the performance achieved by the hybrid solution is sufficiently satisfactory.

## 4. Conclusions and Future Work

In this paper, we propose three deep learning based solutions to forecast the flow of maritime vessels. To apply the deep learning approach, we first divide the given marine area into M×N grids, then we predict both the inflow and outflow of vessels for each grid. In particular, the three solutions are characterized by a CNN, a LSTM, and the integration of BDLSTM and CNN, respectively. When testing them with the real AIS data of vessels, the hybrid solution based on BDLSTM-CNN achieves the best performance in terms of mean absolute error (MAE) and root mean square error (RMSE), both error rates will be decreased by 1–2% compared with other methods, as it is able to coherently learn the spatial and temporal representations for both the inflow and outflow. On the other hand, when further comparing them with a conventional approach, the three deep learning based solutions significantly outperform the SVR method. We would like to note that, N=7 is only an empirical value for the given area in this paper, and it can vary with different scenarios. Moreover, the number of columns does not need to be the same with the rows.

However, the proposed approach still needs to be improved further and tested more extensively. In future, we will work on the following directions: (1) we will consider an attention model in the BDLSTM-CNN based solution to further improve the performance; (2) we will explore more relevant features, such as weather, date, kinematics and kinetics of ship, variable speeds of ship movement, collision avoidance maneuvers; (3) we will use more than five data points to predict a new one, and also forecast the flow longer-time away, such as $Y_{t}$ = $X_{t+3},X_{t+4},\ldots$; (4) we will investigate multi-agent based methods and reinforcement learning based methods to solve the route planning for maritime vessel flows; (5) we will try to apply integral of multiplied by absolute error (ITAE) and integral of time multiplied by square error (ITSE) performance criteria to assess the quality of vessel traffic forecasting. 

## Figures and Tables

**Table 1:** Comparison for Error Rates (%).

	SVR	CNN	LSTM	BDLSTM-CNN
$Y_{t}=X_{t+1}$, NMAE	50.1	24.5	22.5	22.0
$Y_{t}=X_{t+2}$, NMAE	50.5	26.0	24.0	22.5
$Y_{t}=X_{t+1}$, NRMSE	51.2	24.0	21.0	20.0
$Y_{t}=X_{t+2}$, NRMSE	51.4	26.0	23.5	21.0
